# Supplementary figures and images for: Facilitators and barriers to contraception access and use for Hispanic American adolescent women: An integrative literature review
Source: PLOS Glob Public Health. 2024 Jul 25;4(7):e0003169. doi: 10.1371/journal.pgph.0003169 (PMC11271872; doi:10.1371/journal.pgph.0003169)

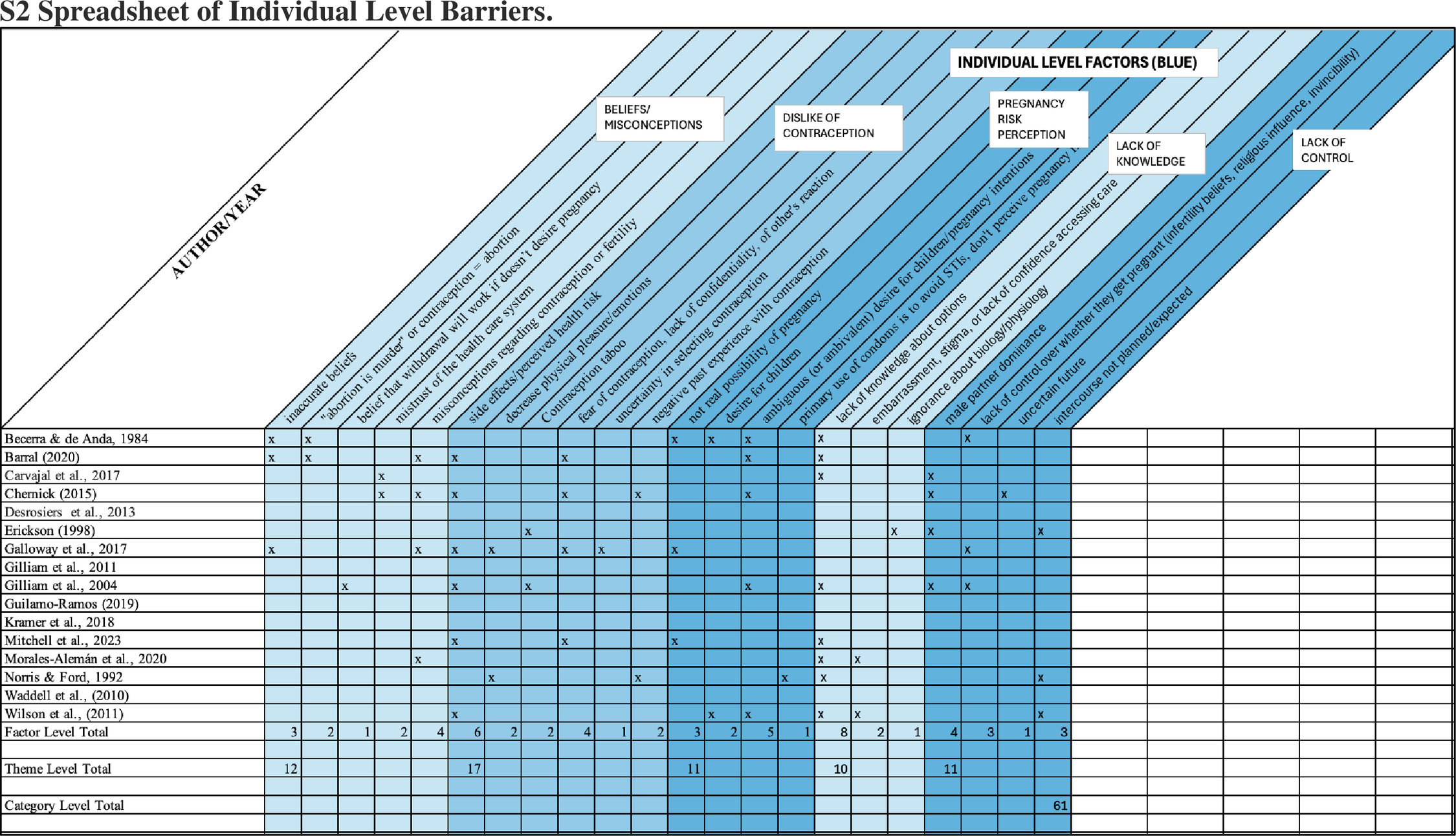

Supplement: S2 Fig — The data sources for the barrier factors are displayed on this spreadsheet. (TIF) [file pgph.0003169.s002.tif]

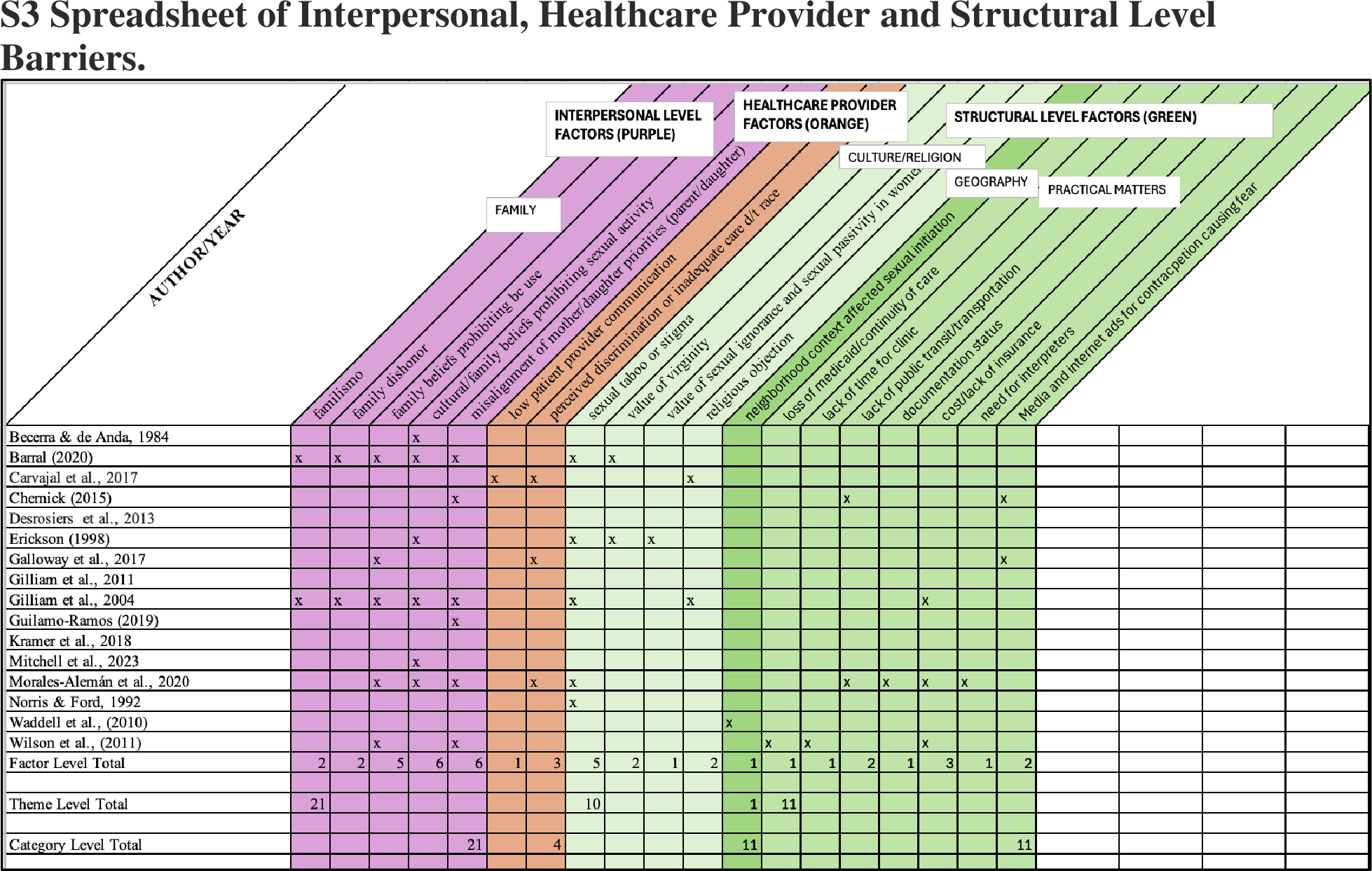

Supplement: S3 Fig — The data sources for the barrier factors are displayed for the interpersonal, healthcare provider and structural level barrier categories. (TIF) [file pgph.0003169.s003.tif]

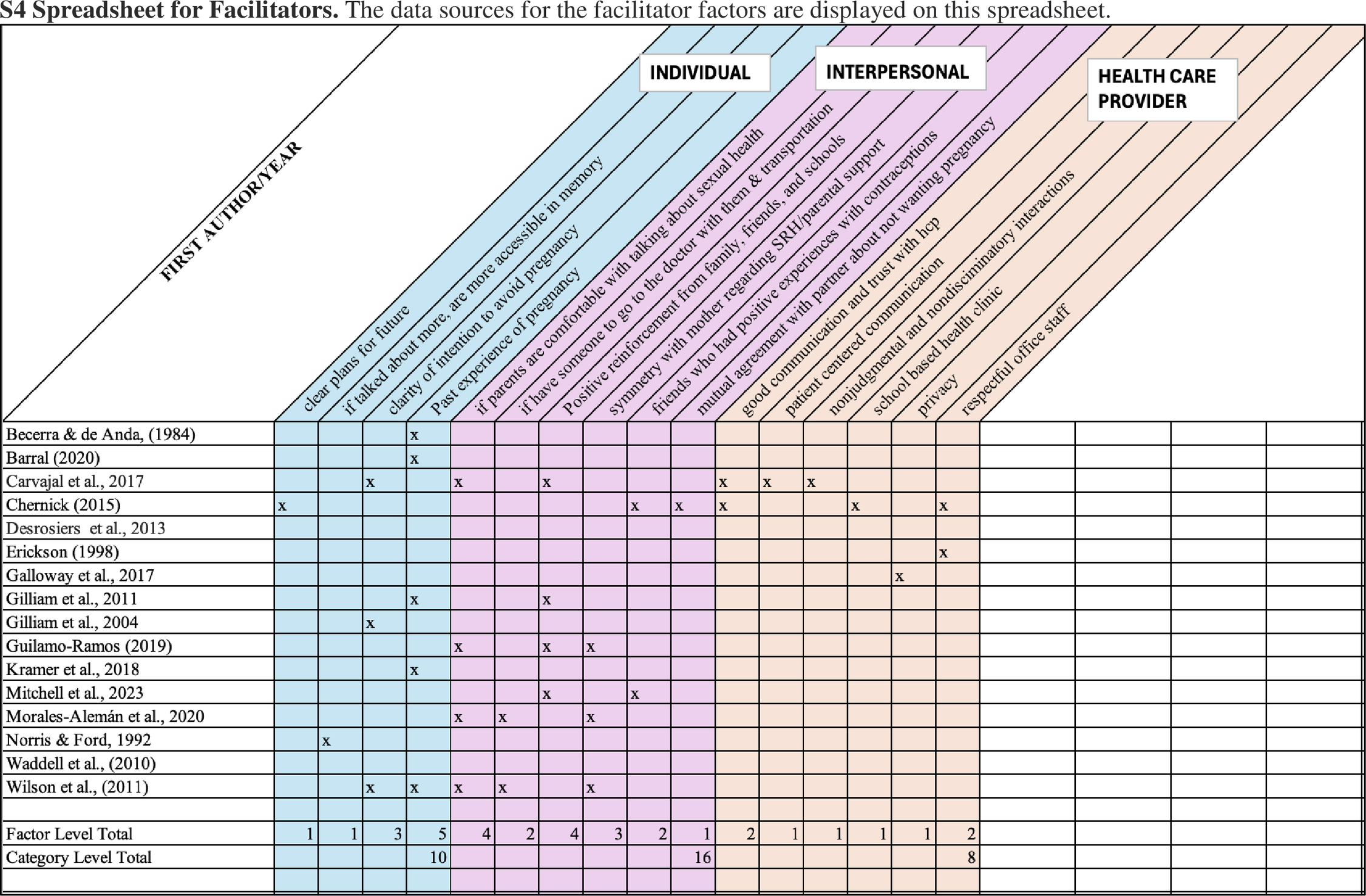

Supplement: S4 Fig — The data sources for the facilitator factors are displayed on this spreadsheet. (TIF) [file pgph.0003169.s004.tif]
